# The Functions of Prospection – Variations in Health and Disease

**DOI:** 10.3389/fpsyg.2018.02328

**Published:** 2018-11-27

**Authors:** Adam Bulley, Muireann Irish

**Affiliations:** ^1^Centre for Psychology and Evolution, School of Psychology, The University of Queensland, St. Lucia, QLD, Australia; ^2^The University of Sydney, Brain and Mind Centre, School of Psychology, Sydney, NSW, Australia; ^3^Australian Research Council Centre of Excellence in Cognition and its Disorders, Sydney, NSW, Australia

**Keywords:** episodic future thinking, episodic foresight, decision-making, hippocampus, prefrontal cortex, Alzheimer’s disease, frontotemporal dementia, evolution

## Abstract

Much of human life revolves around anticipating and planning for the future. It has become increasingly clear that this capacity for *prospective cognition* is a core adaptive function of the mind. Here, we review the role of prospection in two key functional domains: goal-directed behavior and flexible decision-making. We then survey and categorize variations in prospection, with a particular focus on functional impact in clinical psychological conditions and neurological disorders. Finally, we suggest avenues for future research into the functions of prospection and the manner in which these functions can shift toward maladaptive outcomes. In doing so, we consider the conceptualization and measurement of prospection, as well as novel approaches to its augmentation in healthy people and managing its alterations in a clinical context.

## Introduction

A core function of the human mind is to predict and prepare for the immediate and distant future. The capacity for future-oriented cognition has been called prospection (Gilbert and Wilson, 2007; Szpunar et al., 2014), an umbrella term that has been used to cover an array of cognitive phenomena from low-level sensory prediction to the creation of long-term plans (Seligman et al., 2016). Here we focus on one form of prospection: *episodic foresight* or *episodic future thinking* – defined as the imagination of personal future scenarios (Atance and O’Neill, 2005; Suddendorf and Moore, 2011; Szpunar et al., 2014)^1^. This topic has spurred robust debate concerning the underlying mechanisms of future-directed control, and its consequences for a multitude of adaptive behaviors.

To date, prospection has been implicated in everyday adaptive functions as diverse as flexible planning, prospective memory, emotion regulation, and deliberate practice (for reviews see Schacter et al., 2017; Suddendorf et al., 2018). In this article, we first appraise two important general functions of prospection: goal-directed behavior and flexible decision-making. We then explore how variation, as observed via individual differences and lifespan changes, as well as mechanistic alterations in psychopathology and in neurodegenerative disease, affect its functions. A theme of our analysis is that changes in prospection can be both adaptive or maladaptive, and discerning between these outcomes remains an important challenge. To this end, we focus on the following key questions: How do alterations in prospection broadly influence its expressions and functions? How can we objectively categorize differences or changes in prospection? And perhaps most importantly in practical terms, how and when do alterations in prospection become clinically relevant? Finally, we explore important future directions, suggest avenues for improving measurement of prospection, and outline novel approaches to its augmentation in healthy people and management in a clinical context. These include prospection training and ‘strategic compensation’ via cognitive offloading.

## Putative Functions of Prospection

First, what is a ‘function’? An evolutionary approach to cognition and behavior views ‘functions’ as the utility that an adaptive cognitive system or behavior affords to reproductive fitness. Alternatively, ‘adaptive’ and ‘functional’ in the clinical literature and elsewhere can refer to (a) contributions to ‘beneficial’ everyday activities, and/or (b) the case where ‘standard’ operations are not impaired (e.g., Mercuri et al., 2016). Here, we focus on two such *current functions* pertinent to the activities of contemporary everyday living and relevant for wellbeing, namely goal-directed behavior and intertemporal decision-making^2^.

### Goal Directed Behavior

One of the most intuitive functional benefits of prospection is in relation to the setting and pursuit of goals, which can be assessed at different levels of analysis. As a reflection of desired or undesired possible future states of the world, *goals* are, by definition, prospective in nature. Goals may result from the simulation of possible outcomes and ascertaining their emotional significance, yet a goal is more than an “affective forecast” (Wilson and Gilbert, 2005) – it is inherently motivational (Pezzulo et al., 2014). Mental simulations of the future in humans tend to cluster around personal goals, suggesting they represent common mechanisms for organizing and driving adaptive behavior (D’Argembeau, 2016; Lehner and D’Argembeau, 2016). As such, the proclivity for humans to engage in self-referential forms of future-oriented thinking when not otherwise engaged by the external environment has been interpreted as an adaptive manifestation of the brain’s “default” mode (Spreng et al., 2009).

Goal-directed behavior ostensibly underpins many important capacities. One notable example is deliberate practice: repeated actions driven by the goal to improve future capacities (Suddendorf et al., 2016). Deliberate practice is critical not only for achieving expert-level performance on specific tasks, but also for acquiring the wide range of abilities necessary for everyday life. Prospection underpins deliberate practice because it enables people to consider their future self as alterable, with abilities or knowledge that are an improvement on the present. This recognition also serves a motivational role by providing a small-scale internal representation of future payoffs. Thus, deliberate practice is just one useful function of having a ‘temporally extended self’ encompassing memories and anticipations alongside a self-referential narrative that guides the continuing accumulation of skill and knowledge for long-term ends (see Conway, 2005; Prebble et al., 2013). Disruption to deliberate practice in adulthood has clear clinical relevance, yet the role of prospection in this regard has received little attention to date. Exploring the development of deliberate practice in children may offer a useful testbed for understanding its alteration and deterioration in adulthood (Suddendorf et al., 2016).

Decades of research have implicated the frontal lobes in supporting goal-directed behavior (Shallice and Burgess, 1991; Duncan and Owen, 2000). One striking example of compromised goal-directed behavior in the context of frontal lobe dysfunction is provided by the behavioral-variant of frontotemporal dementia (bvFTD), a younger-onset dementia syndrome characterized by habitual, perseverative, and stereotypical behaviors due to degeneration of the medial prefrontal cortex. Patients with bvFTD display a marked incapacity to engage in prospective forms of thinking including simulating the future across personal (Irish et al., 2013), and non-personal (Irish et al., 2016) contexts. Patients increasingly become tethered to the present moment, showing highly inflexible and impulsive behavior driven by a need for immediate gratification where rewarding stimuli are concerned (Ahmed et al., 2015; Wong et al., 2018). An apparent lack of regard for the outcomes of such actions is noted, despite patients retaining an awareness of the ill-timed or inappropriate nature of their behavior.

Unsurprisingly, myriad functional domains related to prospection are compromised in bvFTD (see Irish and Piolino, 2016), as is frequently reported in frontal-lobe syndromes (Shallice and Burgess, 1991; Bechara et al., 2000). Notably, prospective memory, i.e., memory to perform intentions after a delay, is adversely impacted across event and time subscales in bvFTD (Kamminga et al., 2014; Dermody et al., 2015) with patients gravitating toward an increasingly present-oriented response style. Moreover, during conditions of minimal cognitive demand designed to elicit mind wandering (O’Callaghan et al., 2015), bvFTD patients display a marked propensity for stimulus-bound thinking, reflecting an increased reliance on the external environment similar to that observed in ‘environmental dependency syndrome’ (O’Callaghan et al., 2017).

### Flexible Intertemporal Decision-Making

Because people can imagine specific future scenarios, they often face a conflict between anticipated outcomes and present circumstances. *Intertemporal* trade-offs between immediate and delayed costs and benefits are ubiquitous in everyday life (Loewenstein et al., 2003), spanning routine decisions about what to eat for lunch (enjoy the snack, or adhere to one’s diet?) to more profound concerns regarding whom one should marry (perhaps better prospects lie on the horizon?) In laboratory tasks, participants typically make a series of choices between smaller but sooner and larger but later monetary rewards (e.g., $5 now versus $15 in 1 week). Variation in answers to these questions reflects ‘choice impulsivity’ (Gullo et al., 2014; Hamilton et al., 2015), a clinically relevant trait variable which may relate to life expectancy (Bulley and Pepper, 2017), unhealthy behaviors (Story et al., 2014), obesity (Amlung et al., 2016), gambling (Wiehler et al., 2015), and a range of other potentially maladaptive decision-making patterns. It is also exacerbated in various ‘externalizing disorders’ as well in some neurodegenerative disorders (Gleichgerrcht et al., 2010) – for example bvFTD patients show increased delay-discounting, mirroring the prominent displays of impulsivity in their daily lives.

**FIGURE 1:**
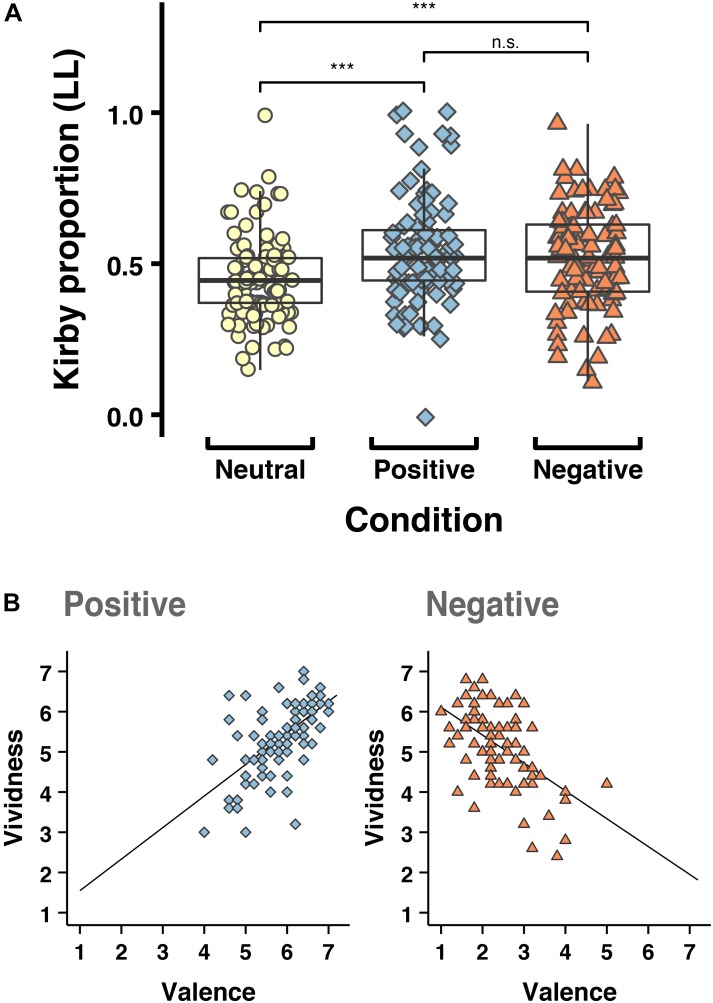
A role for cued prospection in adaptive intertemporal choice? **(A)** From a between-participants study with 297 participants: The mean proportion of larger, later (rather than smaller, sooner) rewards chosen in the Kirby monetary intertemporal choice task when participants were cued with neutral mental imagery (e.g., folding up paper), positive episodic future events (e.g., spending time in nature in 1 week), and negative episodic future events (e.g., getting food poisoning in 1 week). Imagining the future was associated with reduced delay discounting regardless of the valence. ^∗∗∗^ = Significant at *p* < 0.001. **(B)** In the same study, ratings of the event cues demonstrated strong correlations between the vividness with which events were imagined and the emotional impact of those events (valence: 1–7, low scores equate to negative valence and high scores equate to positive valence), illustrating the close ties between episodic mental simulation and emotion. Positive *r* = 0.62, negative *r* = -0.54, *p*’s < 0.001. Figure from Bulley et al. (unpublished).

The capacity to imagine future scenarios allows people to make more prudent, farsighted and flexible decisions that take future consequences – including mutually exclusive possible future outcomes – into account (Gilbert and Wilson, 2007; Boyer, 2008). Accumulating evidence suggests a role for cued episodic foresight in reducing impulsivity (see Figure 1 for a recent example). In a series of recent experiments, participants have been cued to imagine specific, personally relevant future events while they make intertemporal choices or face temptations such as high calorie food (e.g., Dassen et al., 2016). This cuing paradigm consistently reduces choice and behavioral impulsivity: i.e., it makes people more ‘patient’ in their preferences and actions (for reviews see Bulley et al., 2016; Benoit et al., 2018; Rung and Madden, 2018). Such findings dovetail with a growing awareness about the key role of prospection variations in decision-making more broadly (Noël et al., 2017), and underscore the potential utility of prospection in clinical interventions for externalizing disorders.

## Changes in Prospection: Adaptive Alterations Versus Maladaptive Shifts?

We next consider how the dynamic and constructive nature of prospection supports adaptive functional purposes yet may also manifest in maladaptive outcomes (see Henry et al., 2016). Thus, we may ask not only how the mechanisms of prospection deteriorate, but how prospection becomes clinically relevant even when underlying mechanisms are intact. We propose three avenues by which variations in prospection may give rise to adaptive or maladaptive outcomes with a view to stimulating further research in this important area:

### Individual Differences and Shifts in Content

People vary considerably in their tendency to consider the future (Zimbardo et al., 1997), as well as in their preferences for delayed versus immediate rewards (Peters and Büchel, 2011). Such individual differences are important for understanding impulse-related conditions such as addiction, where a prioritization of immediate aspects of a decision-making situation can take precedence (Noël et al., 2017). For example, chronic opiate users have been shown to generate fewer internal (episodic) details when projecting themselves into the future, but not when imagining atemporal scenarios (Mercuri et al., 2016; Moustafa et al., 2018b). The direction of causality here is somewhat opaque, however, given that a disposition to present-orientation may predict the onset of drug use, but chronic drug use may also impact brain functioning – and thus instigate maladaptive feedback loops.

Shifts in the content and modes of episodic future thinking have been documented in detail in affective disorders. Content shifts include an overrepresentation of possible negative future events in both anxiety and depression, while a reduction in the generation of positive future events occurs in depression (for reviews see Miloyan et al., 2014; MacLeod, 2016; Moustafa et al., 2018a). Moreover, subtle shifts in the kinds of details (e.g., episodic versus semantic) and representational format (imagery-based versus verbal-linguistic) of episodic foresight have been demonstrated in various clinical disorders (reviewed in Hallford et al., 2018). We caution, however, against the unilateral labeling of such shifts as ‘impairments,’ as some of these changes may represent coping strategies or adaptive mechanisms for effectively dealing with particular kinds of environmental stressors^3^ (Borkovec et al., 2004; Bulley et al., 2017; Engen and Anderson, 2018). Nevertheless, given that prospection has been implicated in wellbeing in general, it represents an important target for ameliorating distress in clinical populations.

### Mechanistic Impairments

As discussed, neurodegenerative disorders display pervasive changes in prospection, ranging from impaired prospective memory to an inability to envisage and describe the future in rich contextual detail. These compromised capacities reflect distinct underlying patterns of neural degeneration and the breakdown of key cognitive processes known to be important for prospection (Irish et al., 2012c). For example, episodic memory dysfunction precludes episodic future simulation in Alzheimer’s disease (Addis et al., 2009), whereas loss of the conceptual knowledge base represents the key disruptive mechanism in semantic dementia (Irish et al., 2012a,b). Prospection difficulties in Parkinson’s disease, by contrast, are associated exclusively with executive dysfunction (de Vito et al., 2012), while bvFTD represents a more complex picture with multiple neurocognitive processes implicated (Irish et al., 2013). Although the mechanisms by which prospection is altered differ across dementia subtypes, common to all syndromes is the observation of gross functional impairments in activities of daily living. We note, however, that empirical studies definitively linking altered prospection to functional impairment in dementia are lacking and this represents an important area for future investigation (for an initial exploration see Brunette et al., 2018).

### Lifespan Changes

When might a shift in the output of prospection be classified as adaptive? Counter to the prevailing deficit model, we contend that alterations in prospection in healthy aging may serve important adaptive functions (Andrews-Hanna et al., 2018). While older adults produce significantly fewer internal (episodic) details relative to young controls, this is offset by the provision of elevated external (semantic) details (Addis et al., 2010; Abram et al., 2014). This effect likely reflects a shift in the narrative style of older adults wherein overall meaning and context is favored above that of specificity and detail (reviewed by Schacter et al., 2013)^4^.

Older adults also date imagined future events and future self-images much closer to the present time than younger adults (Chessell et al., 2014). This finding has been replicated on naturalistic mind-wandering paradigms with older adults engaging in more atemporal/present-oriented rather than future-oriented spontaneous thoughts (Irish et al., 2018). Such changes make intuitive sense given the increased likelihood of negative events as one nears the end of the lifespan (Chessell et al., 2014). Similarly, worry in older adults shifts to considerations about “family concerns” and “world issues” (for review see Miloyan and Bulley, 2016), and this effect is further apparent in naturally occurring spontaneous thoughts which tend to become less self-focussed (Irish et al., 2018). We tentatively suggest that such alterations in prospection serve a protective function in older age, potentially mediating the well-documented “positivity effect” in healthy aging (Carstensen et al., 2005). When viewed from a functional perspective, the available evidence suggests that the benefits conferred in terms of life outlook and positivity in older adults compensate for their reduction in episodic specificity.

## Future Directions

### Measurement

Given the multifaceted nature of prospection and its diversity of outcomes, how we define and measure it is paramount. The literature is replete with experimental techniques to assess prospection in its many guises. For example, the provision of ‘internal’ (episodic) contextual details is widely used as a marker of the episodic specificity of simulated future events (e.g., Addis et al., 2008), while the number of fulfilled intentions reflects prospective memory capacity (for review see Brandimonte et al., 2014). Miloyan and McFarlane (2018) performed a systematic review of existing episodic foresight tasks, and categorized these measures into six main subcategories: (i) phenomenology (60%); (ii) examination (49%); (iii) fluency (12%); (iv) reaction time (12%); sentence completion (5%); and thought sampling (2%). They concluded that none of the available instruments have been validated to acceptable psychometric standards. An important goal then is to develop appropriate measurement tools that permit the reliable assessment of prospection in clinical settings. The refinement of coding protocols to index the intersection of episodic and semantic elements within future thinking narratives may further offer improved differentiation between clinical syndromes (Strikwerda-Brown et al., 2018), moving beyond a strict episodic-semantic dichotomy when assessing prospection (Irish and Piguet, 2013; Szpunar et al., 2014).

### Toward Enhancement and Treatment

Finally, we briefly consider the pertinent question of how to augment prospection to support everyday function in healthy individuals and to intervene effectively in the context of impairment. We propose two broad categories that hold promise:

#### Training Approaches

There have been numerous attempts to (a) directly improve simulation abilities or to guide the content thereof, and (b) use simulation abilities to augment other functions. The first category includes protocols such as working memory training (Bickel et al., 2011; Hill and Emery, 2013) and episodic specificity induction techniques (Madore et al., 2014) to bolster the provision of episodic detail during prospection. The second category includes the use of future event simulation techniques to improve prospective memory performance (Brewer and Marsh, 2010; Neroni et al., 2014; Altgassen et al., 2015), and to reduce delay discounting (e.g., Peters and Büchel, 2010). The applicability of the above-described approaches to clinical populations, however, remains largely unknown. In severe clinical cases where such interventions are arguably most necessary, it may be particularly difficult to implement simulation training or to leverage prospection to improve other tasks. Moreover, given that prospection is adversely affected in clinical conditions including depression (Williams et al., 1996; Addis et al., 2016), the efficacy and generalizability of such approaches remains an important open question^5^.

#### Strategic Compensation

**FIGURE 2:**
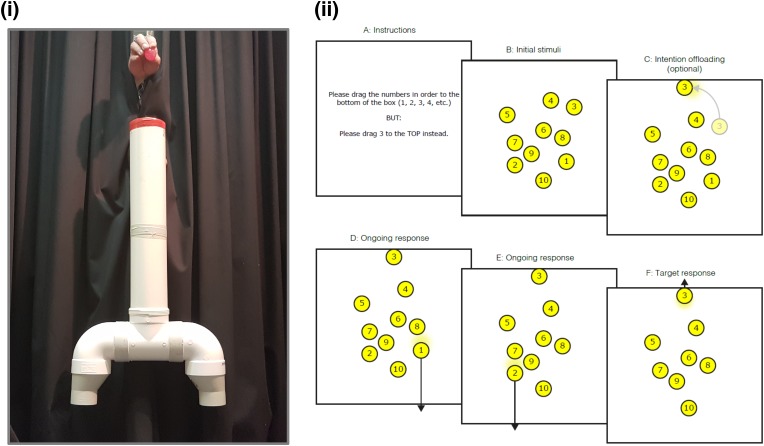
Recent minimalist paradigms for investigating basic mechanisms of prospection. **(i)** Placing one hand under each opening from the forked tube demonstrates a capacity to prepare for two mutually possible future events, thereby demonstrating the rudiments of advanced ‘contingency planning’ (Redshaw and Suddendorf, 2016). **(ii)** In a reminder-setting task, participants drag numbered circles in ascending order to the bottom of the box. They must also remember to carry out either one or three alternative actions for specific numbers (dragging them to a particular edge) **(A,B)**. In some conditions, participants have the option of dragging the target circles to the relevant edge of the box *at the beginning of the trial* – a reminder setting strategy **(C)**. If participants do pursue this option, then – after dragging non-target circles to the bottom of the box **(D,E)** – the new location of the target circles will remind them of the required action **(F)** (Redshaw et al., 2018). Child Development © 2018 Society for Research in Child Development, Inc. All rights reserved. 0009-3920/2018/8906-0015.

Metacognitive insight enables people to appreciate that their simulations of the future ‘could be wrong’. This insight allows people to amend and update their expectations as appropriate, as well as to perform various strategic behaviors to compensate for prospection failures (Redshaw and Bulley, 2018). Two prominent examples are *contingency planning* and *cognitive offloading*:

(a) Contingency planning for mutually exclusive possible outcomes is a complex ability that requires the insight that one’s representations of the future could be incorrect. Contingency planning is critical for numerous functions in everyday life, from arranging insurance and keeping receipts, to planning alternative transport options for important appointments; and from packing an umbrella in case it rains to backing-up one’s hard-drive in case it gets corrupted. Fundamentals of contingency planning for mutually exclusive future events have been studied in child development and in other animals (e.g., Redshaw and Suddendorf, 2016), but its application in clinical settings has yet to receive concerted attention. Nonetheless, we note that some of the non-verbal protocols stemming from developmental and comparative psychology hold potential for translation into clinical populations characterized by cognitive impairment (see Figure 2, panel (i) for a recent example of a paradigm for exploring the capacity to prepare for mutually exclusive future events).

(b) Humans frequently set reminders, write lists, and modify their present surroundings in a variety of ways to augment future cognitive performance. With the increasing ubiquity of technologies that permit future-directed *cognitive offloading* in the form of calendars, alarms, and digital personal assistants, such strategies represent promising forms of intervention in clinical settings (see Figure 2, panel (ii) for a recently developed minimalistic paradigm to examine cognitive offloading). Cognitive offloading likely requires metacognitive insight into the limits of one’s own future performance in order for successful pre-emptive compensation (Risko and Gilbert, 2016), and thus may be most suitable as an intervention opportunity in clinical populations where an awareness of disorder-related limitations remains intact.

## Conclusion

Prospection is a multifaceted construct, which supports a diverse range of important functions including goal-directed behavior and flexible decision-making. Our brief survey of the extant literature, focussing on episodic future thinking, highlights the manifold expressions of prospection and how its functional outcomes can vary according to individual differences (e.g., addiction), lifespan changes (e.g., healthy aging), and disruption of underlying neurocognitive mechanisms (e.g., dementia). We suggest that this inherent variability in the outcomes of prospection may serve important adaptive functions as exemplified in healthy aging. Perhaps most importantly, we note the potential for shifts in content that give rise to maladaptive expressions of prospection even when the underlying mechanisms appear to be in working order or even augmented (e.g., anxiety). A precise understanding of the contributing factors that predispose maladaptive expressions of prospection remains unclear, yet will be critical to inform targeted behavioral interventions. Our intention here is to stimulate further research into the potential for simulation-based training and ‘strategic compensation’ strategies to explore the fundamentals of prospection in clinical contexts and ultimately improve wellbeing in everyday life.

## Author Contributions

AB and MI contributed equally to the conceptualization, literature review, and writing of this manuscript.

## Conflict of Interest Statement

The authors declare that the research was conducted in the absence of any commercial or financial relationships that could be construed as a potential conflict of interest. The reviewer GR declared a shared affiliation, with no collaboration, with one of the authors AB to the handling Editor at the time of the review.

## Footnotes

1 Relevant reviews of prospection and its measurement in the context of ‘low-level’ sensory prediction and reinforcement learning can be found in Friston (2009), Bar (2011), Bubić and Abraham (2014), Clark (2015), and Pezzulo (2016).

2 Note that the evolutionary “functions” and “function” in the clinical sense may in some circumstances converge, but they need not. Evolutionary processes create systems that maximize inclusive fitness, and while wellbeing is often a proxy for the successful operation of these functions, the two frequently diverge (Nettle, 2005; von Hippel, 2018).

3 The capacity to imagine virtually any possible future threat event and generate anxiety before any cues of danger arise is of obvious evolutionary advantage (Miloyan et al., 2018). Even in contemporary environments, it may motivate people to take precautionary steps to avoid danger (Marks and Nesse, 1994; Nesse, 2011). However, threat prospection is also a potent source of distress, to the point of being a core diagnostic feature of anxiety disorders such as GAD. This case illustrates the nuances of addressing the potential ‘functionality’ of prospection variation in clinical contexts.

4 Note that in developmental psychology the focus of research has been less on the content or format of prospection, and instead has concerned the fundamentals of the capacity itself: i.e., what is the developmental trajectory of future-directed cognition and when do the relevant subcomponents ‘come online’? (for reviews see Atance, 2015; Suddendorf and Redshaw, 2013; Suddendorf, 2017).

5 Note that there have also been some calls to directly target prospection in depression with ‘future directed’ therapies (Vilhauer et al., 2012; Roepke and Seligman, 2015).
